# Interobserver Agreement in Automatic Segmentation Annotation of Prostate Magnetic Resonance Imaging

**DOI:** 10.3390/bioengineering10121340

**Published:** 2023-11-21

**Authors:** Liang Jin, Zhuangxuan Ma, Haiqing Li, Feng Gao, Pan Gao, Nan Yang, Dechun Li, Ming Li, Daoying Geng

**Affiliations:** 1 Radiology Department, Huashan Hospital, Affiliated with Fudan University, Shanghai 200040, China; jin_liang@fudan.edu.cn (L.J.); lihaiqing@fudan.edu.cn (H.L.); 2 Radiology Department, Huadong Hospital, Affiliated with Fudan University, Shanghai 200040, China; zxma21@m.fudan.edu.cn (Z.M.); gaofenga1@126.com (F.G.); 15620935261@163.com (P.G.); yn17765505080@163.com (N.Y.); 22211280034@m.fudan.edu.cn (D.L.); 3 Institute of Functional and Molecular Medical Imaging, Shanghai 200040, China

**Keywords:** prostate, radiomics, interobserver agreement, automatic segmentation, T2-weighted imaging

## Abstract

We aimed to compare the performance and interobserver agreement of radiologists manually segmenting images or those assisted by automatic segmentation. We further aimed to reduce interobserver variability and improve the consistency of radiomics features. This retrospective study included 327 patients diagnosed with prostate cancer from September 2016 to June 2018; images from 228 patients were used for automatic segmentation construction, and images from the remaining 99 were used for testing. First, four radiologists with varying experience levels retrospectively segmented 99 axial prostate images manually using T2-weighted fat-suppressed magnetic resonance imaging. Automatic segmentation was performed after 2 weeks. The Pyradiomics software package v3.1.0 was used to extract the texture features. The Dice coefficient and intraclass correlation coefficient (ICC) were used to evaluate segmentation performance and the interobserver consistency of prostate radiomics. The Wilcoxon rank sum test was used to compare the paired samples, with the significance level set at *p* < 0.05. The Dice coefficient was used to accurately measure the spatial overlap of manually delineated images. In all the 99 prostate segmentation result columns, the manual and automatic segmentation results of the senior group were significantly better than those of the junior group (*p* < 0.05). Automatic segmentation was more consistent than manual segmentation (*p* < 0.05), and the average ICC reached >0.85. The automatic segmentation annotation performance of junior radiologists was similar to that of senior radiologists performing manual segmentation. The ICC of radiomics features increased to excellent consistency (0.925 [0.888~0.950]). Automatic segmentation annotation provided better results than manual segmentation by radiologists. Our findings indicate that automatic segmentation annotation helps reduce variability in the perception and interpretation between radiologists with different experience levels and ensures the stability of radiomics features.

## 1. Introduction

Prostate cancer is the second leading cause of cancer-related death in men; in 2021, the official cancer statistics estimated there would be 248,530 (26%) new cases of prostate cancer [1]. Many studies have contributed to the early screening and detection of prostate cancer [2,3,4]. Artificial intelligence (AI)-based techniques, such as deep learning and machine learning, have made the evaluation of the screening and diagnosis, prediction of aggressiveness, and prognosis of prostate cancer faster and more accurate in recent years [2,3,4,5].

Prostate segmentation is a critical step in the automated detection or classification of prostate cancer using AI algorithms [6,7,8]. Accurate manual segmentation in medical imaging is a labor-intensive task and should be conducted by radiologists or physicians with extensive experience as radiologists with various levels of experience differ in their recognition of organ boundaries, especially on medical images of small organs or lesions. Two recent studies exploring the methods of eliminating interobserver variation [9,10] invited senior radiologists to serve as quality control after initial manual segmentation by junior or less-experienced radiologists and found that interobserver variation still existed among the senior radiologists following this workflow.

Many approaches have been proposed to implement automatic or fully automated segmentation in medical imaging to improve accuracy in an efficient manner. Moreover, automatic segmentation has become popular and freely accessible with the MONAI framework, which is an open source platform for deep learning in medical imaging [11]. Herein, we developed an automatic segmentation-based workflow for prostate segmentation using T2-weighted fat saturation images and aimed to investigate whether this type of AI-based workflow eliminates consistency differences among radiologists.

## 2. Materials and Methods

### 2.1. Study Population

**Figure 1:**
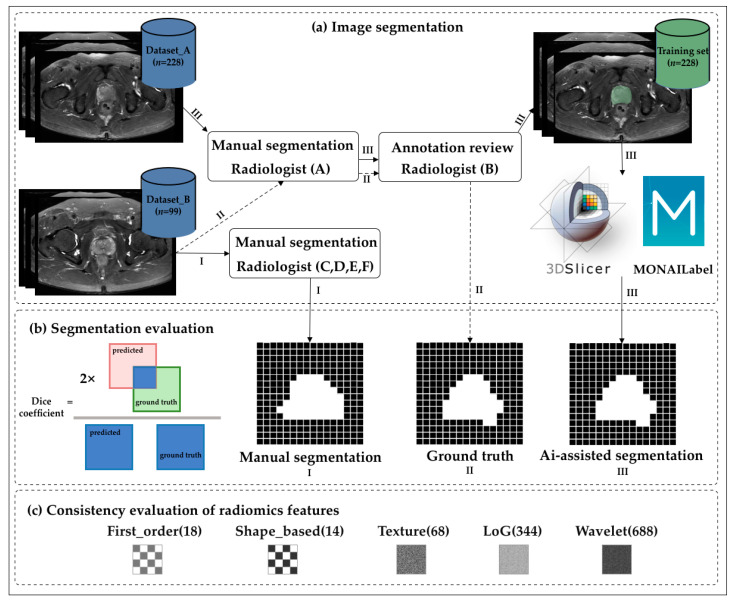
Flowchart of the study. (**a**) The transverse axial fast spin echo T2-weighted images of the prostate were segmented manually and automatically by radiologists with different levels of experience. (**b**) The Dice coefficient was used to accurately measure the spatial overlap of manually delineated images. (**c**) The intraclass correlation coefficient was used to evaluate the stability of the radiomics features. The numbers in the figure indicate the number of features.

This retrospective study was approved by the Institutional Review Board of our hospital and conducted in accordance with the tenets of the Helsinki Declaration of 1975 (revised in 2013). The requirement for informed consent was waived owing to the retrospective study design. Samples were collected from patients with prostate cancer who underwent magnetic resonance imaging (MRI) examination at our hospital between September 2016 and June 2018. The inclusion criteria were (1) preoperative MRI examination and (2) no prostate biopsy, surgery, radiotherapy, or endocrine therapy performed before the MRI examination. Patients who had undergone catheter placement or previous treatment for prostate cancer and exhibited artifacts on MRI were excluded from this study (Figure 1).

### 2.2. Dataset Description and MR Image Acquisition

All the images were obtained on a 3T MR System (MAGNETOM Skyra, Siemens Healthcare, Erlangen, Germany) using a standard 18-channel phased array body coil and a 32-channel integrated spine coil. Patients were given a small amount of food the day before the examination, after which they fasted for 4–6 h and then emptied their bowel and bladder as much as possible prior to the examination. During the examination, the coil was secured with a band to minimize motion artifacts attributed to the patient’s breathing. Axial T2-weighted fast spin-echo (T2FSE) imaging was performed with a slice thickness of 5–7 mm, no spacing, and a field of view of 12 × 12 cm^2^, including the entire prostate and seminal vesicle. All the MRI data were divided into two datasets, namely Dataset A for automatic segmentation training and Dataset B for testing. Datasets A and B contained T2FS images of 228 patients and 99 patients with prostate cancer, respectively (Table 1).

### 2.3. Manual Segmentation

Six radiologists from two centers annotated the prostate on T2FS imaging. Two chief radiologists (Radiologists A and B) with more than 15 years of experience in abdominopelvic diagnosis conducted the manual segmentation, which was used as the reference. All T2FS images from all 327 patients were first manually segmented by Radiologist A at one medical center and then confirmed by Radiologist B at the second medical center. Disagreements were resolved by discussion until consensus was reached.

After confirmation using the reference, two senior radiologists with more than 8 years of experience (Radiologists C and D) and two junior radiologists with 3 years of residency training in radiology (Radiologists E and F) from the second center completed the segmentation of 99 patients (Dataset B), first manually and 2 weeks later assisted by the automatic segmentation model. Manual segmentation of the prostate in all the T2FS images was performed using open-source software (3D Slicer, version 4.8.1; National Institutes of Health; https://www.slicer.org).

### 2.4. Automatic Computer-Aided Segmentation

**Figure 2:**
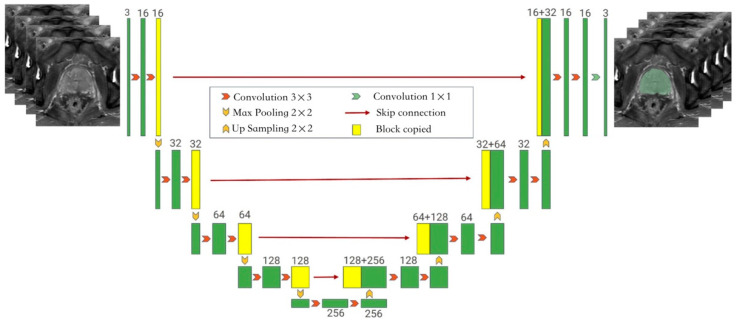
The framework of the auto-segmentation model showing the Monai-3D-UNet structure proposed in this paper for prostate segmentation. The left side of the network acts as an encoder to extract features at different levels, while the right side acts as a decoder to aggregate features and segment masks. In the coding phase, the encoder extracts features from multiple scales and generates a fine-to-rough feature map. Fine feature maps contain lower-level features but more spatial information, while rough feature maps provide the opposite information. In the coding stage, the input is a 128 × 128 × 128 three-channel voxel and the output is a 128 × 128 × 128 voxel. Each layer of the coding part contains two 3 × 3 × 3 convolutions. After the convolution layer, BN + ReLU is used to activate the function, and then 2 × 2 × 2 max pooling is added. The stride is 2. In the decoding section, each layer has a 2 × 2 × 2 upper convolution operation with a stride of 2, followed by two 3 × 3 × 3 convolution and BN + ReLU activation functions. In the final layer, the 1 × 1 × 1 convolution reduces the number of output channels to the number of labels and uses Softmax as a loss function.

MONAI_Label is a free, intelligent, open-source image annotation and learning tool. Modules that work with 3DSlicer enable users to create annotated datasets and build AI-based annotation models for clinical evaluation. To overcome the potential bias among the small number of readers, we developed a prostate-assisted segmentation model on the 3DSlicer platform based on MONAI_Label and trained it exclusively using Dataset A (Figure 2). In practical application scenarios, sufficient and high-quality datasets with high fidelity are usually not available, and the acquisition of perfect datasets becomes particularly challenging. Therefore, we utilized data augmentation to reduce the reliance on training data, thereby aiding the development of AI models with high accuracy and better speed. The model uses MONAI_Label’s built-in API to flip, rotate, crop, scale, translate, and shake the image to expand the training samples.

### 2.5. Data Analysis and Statistical Methods

#### Evaluation of the Segmentation Model

All the statistical analyses were performed using R (version 3.6.3), Python (version 3.9.7), and SPSS (version 22). The Wilcoxon rank sum test was used to compare the paired samples. The statistical significance level was set at *p* < 0.05. In the interobserver cohort, the Dice coefficient was used as a measure of the accuracy of the spatial overlap of the manually delineated images. A Dice coefficient of 0 indicates no overlap, and that of 1 indicates exact overlap.

### 2.6. Consistency Evaluation of the Radiomics Features

Pyradiomics was used to extract the radiomics features of Dataset B. We analyzed the first-order, texture, Laplacian of Gaussian (LoG), morphological, and wavelet features extracted using the Pyradiomics software package. The intraclass correlation coefficient (ICC) was used to evaluate feature stability; an ICC of 0.75–0.89 was considered good, and an ICC of >0.90 was considered to have excellent reproducibility [12]. Finally, we analyzed the consistency of feature extraction following manual segmentation by different radiologists, as well as after automatic segmentation.

## 3. Results

### 3.1. Evaluation of the Automatic Segmentation Model

The auto-segmentation model was trained using T2FS scans from Dataset A, consisting of images from 228 patients with prostate cancer with a mean age of 69 years. In the present model, 80% of the data were used for training and 20% were used for validation. Thus, 183 patients constituted the training set and the remaining 45 constituted the validation set. A total of 99 scans (mean age: 79 years) from Dataset B were collected for the testing set. Table 1 lists the basic characteristics of the population included in this experiment.

The learning rate of the model was 1 × 10^−4^, the batch size was equal to 1, and the Adam optimizer was used. The model was trained for 300 epochs on a server with an image processor. The random affine transformation was used for data augmentation. The training results of the automatic segmentation model are shown in Table 2. Similarly, we used the nnUNet model for our data training (see Table 2 for the results). Overall, the average Dice coefficient of the auto-segmentation model in the testing set was 0.831. The mean Dice values of the two segmentation models were higher (Dice coefficients > 0.9) in the training dataset; the Dice coefficients of the two segmentation models decreased in the testing set.

### 3.2. Evaluation of the Consistency of Image Segmentation by the Radiologists

**Figure 3:**
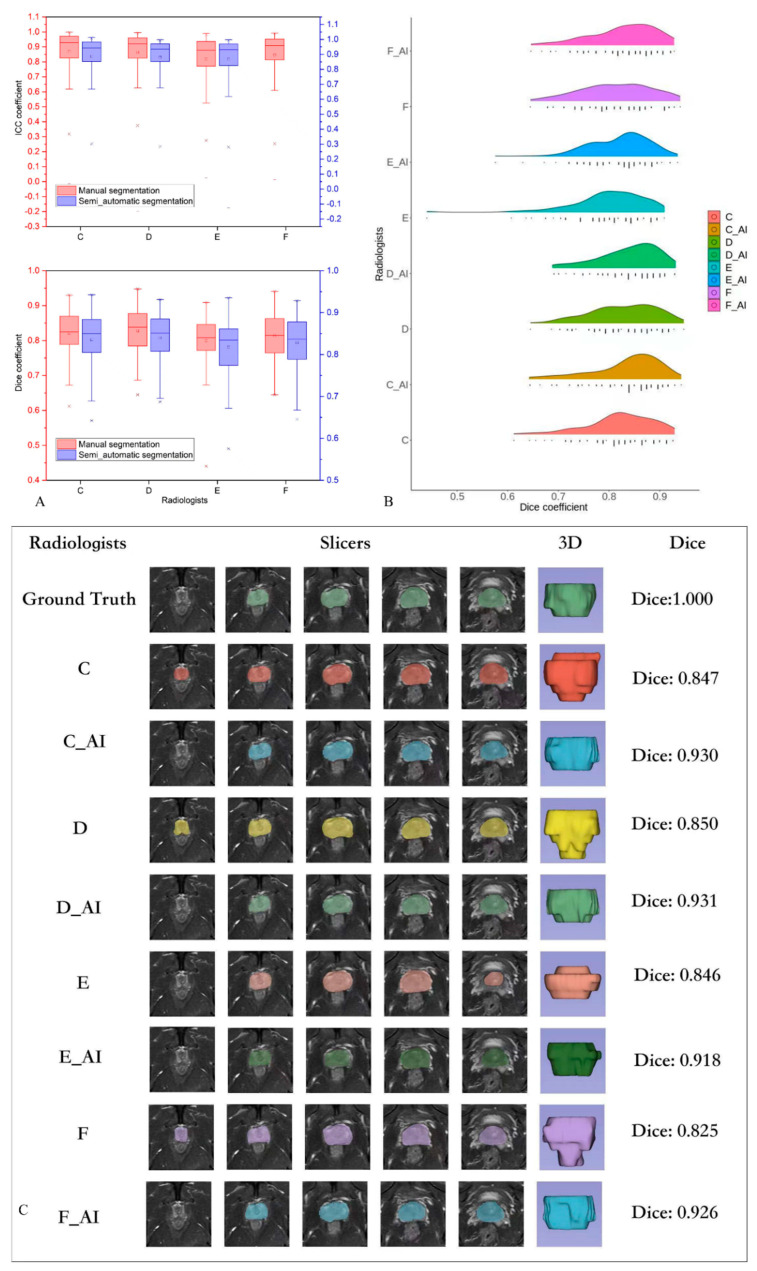
(**A**) Box plot of the segmentation performance of four radiologists for the testing set. (**B**) Performance of all the radiologists: automatic segmentation annotation versus manual segmentation. (**C**) One sample of a radiologist’s performance and their automatic segmentation annotation.

Table 3 presents the segmentation performance of the four radiologists on the testing set. In all 99 columns, the manual and automatic segmentation results of the senior group were significantly better than those of the junior group (*p* < 0.05). Figure 3A shows the box plot of the segmentation performance of the four radiologists on the testing set. The Dice coefficient was higher for all the radiologists using the automatic segmentation than for those performing manual segmentation, except for one senior radiologist who showed no significant improvement (*p* = 0.06). Similarly, we carefully divided the patients with prostate cancer in the testing set into low-grade groups (Grades 1 and 2) and high-grade groups (Grades 3, 4, and 5); however, no significant differences were observed in the manual and automatic labeling between the low- and high-grade group by the radiologists (*p* > 0.05). In the low-grade group, the model did not significantly improve the manual segmentation results (*p* > 0.05). The performance of junior radiologists did not significantly differ from that of both senior radiologists after automatic segmentation annotation (Table 4).

### 3.3. Evaluation of the Segmentation Model

Table 3 shows the consistency of the radiomics features extracted from the segmentation results from four radiologists. Consistency was higher for automatic segmentation images than for manual segmentation images (*p* < 0.05), and the average ICC reached >0.85. Figure 3A shows the box plot of the segmentation agreement between the four radiologists in the testing set.

### 3.4. Consistency Evaluation of the Radiomics Features

Among the five groups of radiomics features (Table 5), the first-order features showed better performance and the shape based features showed worse performance in the “excellent” ICC group. Overall, the number of “excellent” ICC features was lower in the manual segmentation group than in the automatic segmentation group.

## 4. Discussion

In this study, our trained automatic segmentation model demonstrated a median Dice coefficient of 0.850 (0.568, 0.940), as well as high efficiency (11 h) on T2FS imaging compared with nnUNet (median Dice coefficient 0.848 [0.667, 0.911]), which took 125 h. This automatic segmentation model is essentially a standard convolutional neural network (i.e., UNet) [11,13,14]. The performance metrics of this model were better than those of all the manual segmentations performed by the radiologists in this study. The performance of our model was close to the segmentation performance of senior radiologists following the assisted annotation, thereby suggesting the use of our model as a tool supporting the clinicians’ workflow for accurate diagnosis of prostate cancer.

Moreover, consistency significantly improved after automatic segmentation compared with manual segmentation, and the ICC of senior radiologists following automatic segmentation increased to perfect consistency (0.925 [0.888~0.950], Table 6). None of these findings have been reported in previous studies. In our sample, the Dice coefficient and ICC of both senior and junior radiologists significantly improved after automatic segmentation compared with manual segmentation. The Dice coefficients in groups of Grades 3, 4, and 5 in Dataset B (n = 61) of the three radiologists significantly differed between the automatic segmentation annotation and manual segmentation. Our findings indicate that the difficulty in segmenting the prostate in the higher-grade group may have resulted in more variability in manual segmentation compared with the lower-grade group.

This study demonstrates that the performance of junior radiologists following automatic segmentation was similar to that of senior radiologists, while the performance of one junior radiologist was significantly worse compared with that of senior radiologists performing manual segmentation. This indicates that automatic segmentation annotation could reduce the variability in the perception of radiologists with different levels of experience, who may provide different interpretations or ratings [15,16,17], which is a critical issue in image segmentation (Table 4). However, our automatic segmentation annotation procedure could not eliminate the variability in perception and interpretation between junior and senior radiologists. We found a significant difference in the Dice coefficients between junior and senior radiologists even after automatic segmentation annotation, indicating that radiologists may still differ in their perception and interpretation of the prediction area of the automatic segmentation model based on their own experience (Figure 3). AI-based medical imaging analysis usually requires senior radiologists or physicians to manually segment or confirm the structures on medical images as the reference standard or ground truth [18]. Manual segmentation is a labor-intensive and time-consuming task for senior physicians, and it is expensive to hire more than one senior physician for large data-based studies. Our findings suggest that the auto-segmentation model can efficiently accomplish this type of image segmentation following confirmation by senior physicians.

Radiomics features are not stable between the region of interest sizes and volumes on computed tomography and MRI, which was reported in a study using a homogenous phantom without any texture differences [19,20]. Ensuring the stability of radiomics features is crucial for the accuracy of image-based prognostication and external generalization of prognostic models [21,22,23,24,25,26,27,28,29,30,31]. In this study, ICCs were used to evaluate the repeatability and reproducibility of the radiomics features. A MONAI_Label-based automatic prostate segmentation system was established to help guide the selection of stable radiomics features. Our results show that the ICC for all the features increased to ≥0.9 after auto-segmentation-assisted annotation, indicating that the auto-segmentation model can help reduce the segmentation variance among different radiologists and thereby greatly improve the number of reproducible the prostate radiomics features (Table 4).

### Limitations

This study has certain limitations. First, this was a single-institution retrospective study with a limited number of patients; thus, it may not be representative of other institutions. However, the size of our cohort is very similar to other cohorts reported in the literature, which highlights the urgent need for radiomics studies with larger cohorts. In addition, this study focused on prostate cancer; thus, its applicability to other tumor sites has not been demonstrated. Despite the validation of the PyRadiomics platform, the results may differ from those with other radiomics feature extraction platforms. Finally, although we used ICC classification cutoffs commonly used in the literature (0.75 and 0.9) [12], these may not be ideal thresholds for feature inclusion in a prognostic model. Thus, clinically relevant thresholds for the future development of radiomics signature biomarkers for prostate cancer are unclear. Whether the repeatability of individual features significantly impacts the overall performance of a prediction model combining multiple features remains to be further investigated. Despite its limitations, our study systematically assessed the reproducibility of MRI-based radiomics in patients with prostate cancer, which has rarely been performed. Future research should analyze the correlation between radiomics features and clinical variables to reveal the most suitable radiomics features that should be included in prognostic models.

## 5. Conclusions

Our proposed auto-segmentation model exhibited better performance than radiologists performing manual segmentation, and automatic segmentation annotation was better than manual segmentation. Automatic segmentation annotation improved the workflow of image segmentation and reduced the variability in the perception and interpretation of radiologists with different degrees of experience. Furthermore, auto-segmentation-assisted annotation helps ensure the stability of the radiomics features. A large-scale dataset of a multi-center study may help extrapolate the results obtained in this study.

## Figures and Tables

**Table 1:** Clinical characteristics of the patients in Datasets A and B.

Characteristics	Dataset A (*n* = 228)	Dataset B (*n* = 99)
Age (years)	69.0 [65.0–74.0]	70.0 [64.0–75.0]
Grade 1	51 (22.37%)	12 (12.12%)
Grade 2	49 (21.49%)	26 (26.26%)
Grade 3	48 (21.05%)	17 (17.17%)
Grade 4	30 (13.16%)	27 (27.27%)
Grade 5	50 (21.93%)	17 (17.17%)

Variables are expressed as median [interquartile ranges] or count (percentage). Grade 1 (Gleason score < 6): Only individual discrete well-formed glands; Grade 2 (Gleason score 3 + 4 = 7): Predominantly well-formed glands with lesser component of poorly formed/fused/cribriform glands; Grade 3 (Gleason score 4 + 3 = 7): Predominantly poorly formed/fused/cribriform glands with lesser component of well-formed glands; Grade 4 (Gleason score 4 + 4 = 8; 3 + 5 = 8; 5 + 3 = 8): (1) Only poorly formed/fused/cribriform glands, (2) predominantly well-formed glands and lesser component lacking glands, or (3) predominantly lacking glands and lesser component of well-formed glands; and Grade 5 (Gleason scores 9–10): Lacks gland formation (or with necrosis) with or without poorly formed/fused/cribriform glands [1].

**Table 2:** Performance of the segmentation model in the training, validation, and testing sets.

Model	Auto Segmentation Model (MONAI_Label Based)	nnUnet
Training set (n = 183)		
Average Dice (95% CI)	0.918 (0.908, 0.928)	0.947 (0.944, 0.950)
Median Dice [Interquartile Ranges]	0.950 [0.911, 0.967]	0.963 [0.936, 0.977]
Verification set (n = 45)		
Average Dice (95% CI)	0.839 (0.833, 0.844)	0.862 (0.839–0.866)
Median Dice [Interquartile Ranges]	0.854 [0.844, 0.859]	0.884 [0.807, 0.930]
Testing set (n = 99)		
Average Dice (95% CI)	0.831 (0.816, 0.845)	0.838 (0.8279–0.8487)
Median Dice [Interquartile Ranges]	0.850 [0.568, 0.940]	0.848 [0.667, 0.911]
Training time (h)	11	125

CI, confidence interval.

**Table 3:** Dice coefficient and ICC of image segmentation by radiologists with different levels of seniority.

Dataset		All Cases in Dataset B (n = 99)											
		Dice Coefficient in All the Cases in Dataset B			Dice Coefficient in Grades 1 and 2 in Dataset B (n = 38)			Dice Coefficient in Grades 3, 4, and 5 in Dataset B (n = 61)			ICC for Image Segmentation by the Radiologists		
	Radiologist (Time)	Average [CI]	Median (Min, Max)	*p*-Value	Average [CI]	Median (Min, Max)	*p*-Value	Average [CI]	Median (Min, Max)	*p*-Value	Average [CI]	Median (Min, Max)	*p*-Value
Senior radiologists (8 years’ experience)	C (3.67 h)	0.821 [0.808, 0.834]	0.824 (0.61, 0.93)	0.008 **	0.820 [0.800, 0.842]	0.829 (0.612, 0.918)	0.177	0.822 [0.805, 0.839]	0.821 (0.643, 0.930)	0.040 *	0.873 [0.08, 1.0]	0.930 (0.864, 0.881)	<0.01 **
	C_AI (1.98 h)	0.836 [0.822, 0.849]	0.850 (0.64, 0.94)		0.835 [0.810, 0.861]	0.866 (0.642, 0.943)		0.835 [0.820, 0.851]	0.841 (0.646, 0.930)		0.888 [0.10, 1.01]	0.940 (0.880, 0.897)	
	D (3.5 h)	0.828 [0.815, 0.841]	0.838 (0.64, 0.95)	0.049 *	0.829 [0.806, 0.852]	0.829 (0.645, 0.948)	0.416	0.827 [0.812, 0.843]	0.839 (0.686, 0.928)	0.096	0.866 [0.17, 1.0]	0.920 (0.858, 0.875)	<0.01 **
	D_AI (1.56 h)	0.840 [0.828, 0.851]	0.851 (0.69, 0.93)		0.858 [0.817, 0.858]	0.837 (0.687, 0.912)		0.840 [0.826, 0.854]	0.840 (0.695, 0.931)		0.883 [0.11, 1.0]	0.940 (0.875, 0.891)	
Junior radiologists (2 years’ experience)	E (4.5 h)	0.800 [0.785, 0.814]	0.808 (0.44–0.91)	0.005 **	0.830 [0.792, 0.834]	0.813 (0.647–0.899)	0.283	0.791 [0.772, 0.811]	0.796 (0.440–0.909)	0.009 **	0.821 [0.06, 0.99]	0.880 (0.811, 0.831)	<0.01 **
	E_AI (2.3 h)	0.818 [0.805, 0.830]	0.834 (0.58, 0.94)		0.831 [0.794, 0.835]	0.814 (0.575, 0.895)		0.820 [0.804, 0.836]	0.835 (0.630, 0.935)		0.872 [0.14, 1.0]	0.930 (0.863, 0.880)	
	F (5 h)	0.814 [0.800, 0.827]	0.815 (0.64, 0.94)	0.019 *	0.805 [0.792, 0.837]	0.814 (0.674, 0.941)	0.925	0.813 [0.796, 0.831]	0.818 (0.645, 0.930)	0.058	0.848 [0.13, 0.99]	0.910 (0.839, 0.858)	<0.01 **
	F_AI (2.9 h)	0.828 [0.816, 0.841]	0.837 (0.65, 0.93)		0.844 [0.807, 0.849]	0.828 (0.683, 0.929)		0.829 [0.812, 0.845]	0.836 (0.646, 0.926)		0.869 [0.12, 1.0]	0.920 (0.860, 0.878)	

* Wilcoxon rank sum test *p* < 0.05. ICC, intraclass correlation coefficient; CI, confidence interval. ** Wilcoxon rank sum test *p* < 0.01

**Table 4:** Dice comparison between manual and semi-auto segmentation from different radiologists.

Comparison	*p*-Value
C vs. D	0.642
C vs. F	0.759
C vs. E	0.001 **
C vs. C_AI	0.008 *
C vs. D_AI	0.001 **
C vs. F_AI	0.152
C vs. E_AI	0.861
D vs. F	0.119
D vs. E	0.001 **
D vs. C_AI	0.087
D vs. D_AI	0.049 *
D vs. F_AI	0.293
D vs. E_AI	0.618
E vs. F	0.088
E vs. C_AI	0.001 **
E vs. D_AI	0.001 **
E vs. E_AI	0.001 **
E vs. F_AI	0.001 **
F vs. C_AI	0.001 **
F vs. D_AI	0.001 **
F vs. E_AI	0.348
F vs. F_AI	0.009 *
C_AI vs. D_AI	0.567
C_AI vs. F_AI	0.009 *
C_AI vs. E_AI	0.001 **
D_AI vs. F_AI	0.007 *
D_AI vs. E_AI	0.001 **
F_AI vs. E_AI	0.132

* Wilcoxon rank sum test *p* < 0.05. ** Wilcoxon rank sum test *p* < 0.001.

**Table 5:** Dice and 95% Hausdorff distance of 30 random cases of the 325 patients.

Feature Category (n)	Senior Radiologists				Junior Radiologists			
	D	D_AI	C	C_AI	F	F_AI	E	E_AI
First_order (18)								
ICC ≥ 0.9	10 (55.6)	13 (72.2)	9 (50.0)	14 (77.8)	12 (66.7)	13 (72.2)	7 (38.9)	14 (77.8)
0.75 < ICC < 0.9	5 (27.8)	2 (11.1)	6 (33.3)	1 (5.6)	3 (16.7)	2 (11.1)	8 (44.4)	1 (5.6)
ICC ≤ 0.75	3 (16.7)	3 (16.7)	3 (16.7)	3 (16.7)	3 (16.7)	3 (16.7)	3 (16.7)	3 (16.7)
Shape_based (14)								
ICC ≥ 0.9	0 (0.0)	2 (14.3)	0 (0.0)	2 (14.3)	2 (14.3)	0 (0.0)	0 (0.0)	0 (0.0)
0.75 < ICC < 0.9	9 (64.3)	7 (50.0)	8 (57.1)	6 (42.9)	7 (50.0)	8 (57.1)	9 (64.3)	8 (57.1)
ICC ≤ 0.75	5 (35.7)	5 (35.7)	6 (42.9)	6 (42.9)	5 (35.7)	6 (42.9)	5 (35.7)	6 (42.9)
Texture (68)								
ICC ≥ 0.9	37 (54.4)	46 (67.6)	43 (63.2)	48 (70.6)	27 (39.7)	38 (55.9)	19 (27.9)	36 (52.9)
0.75 < ICC < 0.9	22 (32.4)	15 (22.1)	17 (25.0)	13 (19.1)	29 (42.6)	22 (32.4)	33 (48.5)	24 (35.3)
ICC ≤ 0.75	9 (13.2)	7 (10.3)	8 (11.8)	7 (10.3)	12 (17.6)	8 (11.8)	16 (23.5)	8 (11.8)
LoG (344)								
ICC ≥ 0.9	186 (54.1)	212 (61.6)	197 (57.3)	222 (64.5)	181 (52.6)	202 (58.7)	145 (42.2)	218 (63.4)
0.75 < ICC < 0.9	95 (27.6)	82 (23.8)	78 (22.7)	75 (21.8)	85 (24.7)	86 (25.0)	111 (32.3)	79 (23.0)
ICC ≤ 0.75	63 (18.3)	50 (14.5)	63 (18.3)	47 (13.7)	78 (22.7)	56 (16.3)	88 (25.6)	47 (13.7)
Wavelet (688)								
ICC ≥ 0.9	417 (60.6)	465 (67.6)	437 (63.5)	454 (66.0)	395 (57.4)	412 (59.9)	295 (42.9)	400 (58.1)
0.75 < ICC < 0.9	173 (25.1)	130 (18.9)	160 (23.3)	136 (19.8)	189 (27.5)	159 (23.1)	259 (37.6)	178 (25.9)
ICC ≤ 0.75	98 (14.2)	93 (13.5)	91 (13.2)	98 (14.2)	104 (15.1)	117 (17.0)	134 (19.5)	110 (16.0)
All_features (1132)								
ICC ≥ 0.9	650 (57.4)	738 (65.2)	686 (60.6)	740 (65.4)	617 (54.5)	665 (58.7)	466 (41.2)	668 (59.0)
0.75 < ICC < 0.9	304 (26.9)	236 (20.8)	275 (24.3)	231 (20.4)	313 (27.7)	277 (24.5)	420 (37.1)	290 (25.6)
ICC ≤ 0.75	178 (15.7)	158 (14.0)	171 (15.1)	161 (14.2)	202 (17.8)	190 (16.8)	246 (21.7)	174 (15.4)

n, Number of features falling into the excellent (ICC ≥ 0.9), good (0.75 < ICC < 0.9), and other (ICC ≤ 0.75) categories for all the features and distinct feature types (first_order, shape, texture, LoG filtered, and wavelet filtered); ICC, intraclass coefficient.

**Table 6:** Consistency evaluation of segmentation by different radiologists.

Radiologist		Single Measure ICC (C,1)	Mean of k Measurements ICC (C,K)
C	D	0.505 (0.342~0.638)	0.671 (0.510~0.779)
C	E	0.379 (0.197~0.535)	0.549 (0.329~0.697)
C	F	0.493 (0.329~0.629)	0.661 (0.495~0.772)
C	C_AI	0.505 (0.343~0.638)	0.671 (0.510~0.779)
C	D_AI	0.542 (0.273~0.733)	0.703 (0.429~0.846)
C	E_AI	0.560 (0.409~0.682)	0.718 (0.580~0.811)
C	F_AI	0.486 (0.320~0.623)	0.654 (0.485~0.768)
D	E	0.435 (0.260~0.581)	0.606 (0.413~0.735)
D	F	0.299 (0.108~0.468)	0.460 (0.196~0.637)
D	C_AI	0.502 (0.220~0.706)	0.668 (0.361~0.827)
D	D_AI	0.483(0.324~0.625)	0.652(0.490~0.770)
D	E_AI	0.475 (0.307~0.614)	0.644 (0.469~0.761)
D	F_AI	0.425 (0.249~0.574)	0.597 (0.399~0.729)
E	F	0.396 (0.217~0.550)	0.568 (0.356~0.710)
E	C_AI	0.338 (0.199~0.536)	0.505 (0.332~0.698)
E	D_AI	0.318 (0.199~0.536)	0.483 (0.332~0.694)
E	E_AI	0.306 (0.126~0.481)	0.468 (0.225~0.650)
E	F_AI	0.578 (0.321~0.756)	0.733 (0.486~0.861)
F	C_AI	0.413 (0.258~0.579)	0.585 (0.410~0.733)
F	D_AI	0.431 (0.294~0.605)	0.602 (0.455~0.754)
F	E_AI	0.370 (0.061~0.614)	0.540 (0.115~0.761)
F	F_AI	0.483 (0.328~0.628)	0.651 (0.493~0.771)
C_AI	D_AI	0.861 (0.799~0.904)	0.925 (0.888~0.950)
C_AI	E_AI	0.731 (0.623~0.811)	0.844 (0.768~0.895)
C_AI	F_AI	0.751 (0.651~0.826)	0.858 (0.788~0.905)
D_AI	F_AI	0.757 (0.658~0.830)	0.862 (0.794~0.907)
D_AI	E_AI	0.608 (0.467~0.718)	0.756 (0.637~0.836)
F_AI	E_AI	0.699 (0.583~0.787)	0.823 (0.736~0.881)

## Data Availability

All the data will be shared upon reasonable request by the corresponding author.
