# Correction: Jin et al. Interobserver Agreement in Automatic Segmentation Annotation of Prostate Magnetic Resonance Imaging. *Bioengineering* 2023, *10*, 1340

**DOI:** 10.3390/bioengineering11101027

**Published:** 2024-10-15

**Authors:** Liang Jin, Zhuangxuan Ma, Haiqing Li, Feng Gao, Pan Gao, Nan Yang, Dechun Li, Ming Li, Daoying Geng

**Affiliations:** 1 Radiology Department, Huashan Hospital, Affiliated with Fudan University, Shanghai 200040, China; jin_liang@fudan.edu.cn (L.J.); lihaiqing@fudan.edu.cn (H.L.); 2 Radiology Department, Huadong Hospital, Affiliated with Fudan University, Shanghai 200040, China; zxma21@m.fudan.edu.cn (Z.M.); gaofenga1@126.com (F.G.); 15620935261@163.com (P.G.); yn17765505080@163.com (N.Y.); 22211280034@m.fudan.edu.cn (D.L.); 3 Institute of Functional and Molecular Medical Imaging, Shanghai 200040, China

The authors regret to pinpoint two editorial errors in [1], which correction is needed in the sake of clarity, without affecting the general scientific validity. 

**(1) Title of Table 5.**

The correct title is:

Table 5. Consistency results of radiomics features among radiologists with both manual and semi-auto segmentation.

**(2) In Figure 3A (upper box-plot) the ICC box of radiologist F with semi-automatic segmentation (purple) was missing.**

This is the Figure 3 with correct box-plot:

**Figure 3:**
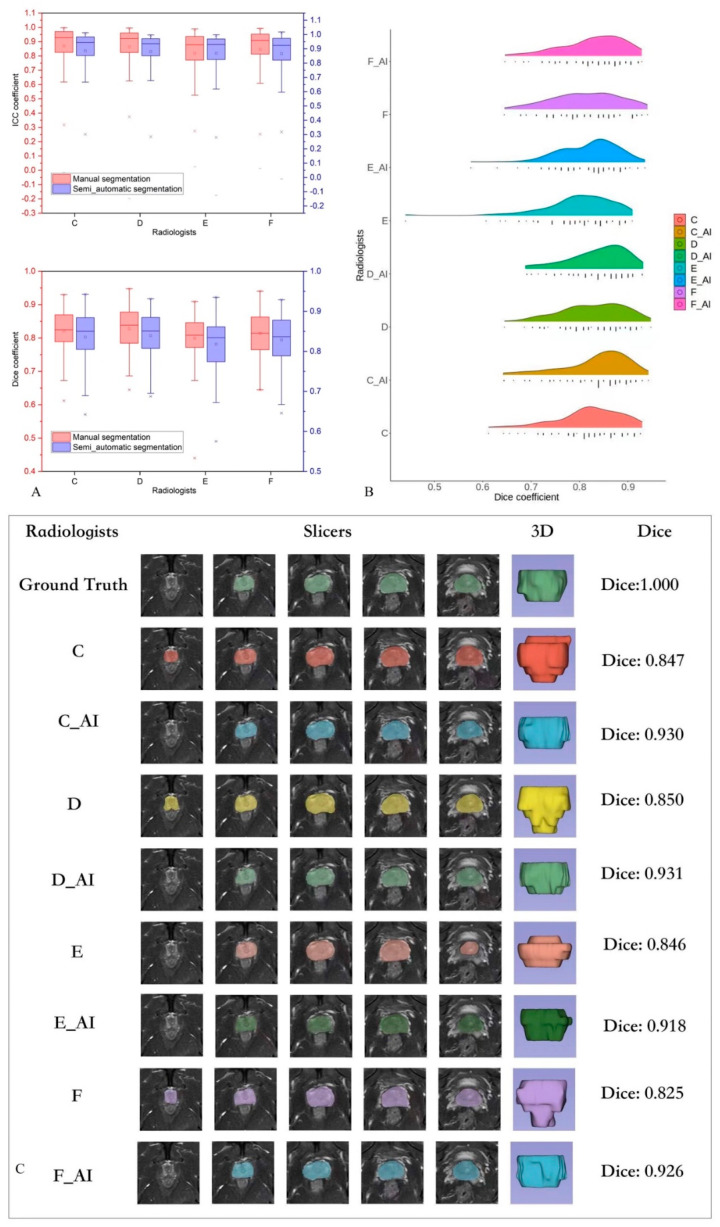
(**A**) Box plot of the segmentation performance of four radiologists for the testing set. (**B**) Performance of all the radiologists: automatic segmentation annotation versus manual segmentation. (**C**) One sample of a radiologist’s performance and their automatic segmentation annotation.
